# Correction to: SBbadger: biochemical reaction networks with definable degree distributions

**DOI:** 10.1093/bioinformatics/btad117

**Published:** 2023-03-09

This is a correction to: Michael A Kochen, H Steven Wiley, Song Feng, Herbert M Sauro, SBbadger: biochemical reaction networks with definable degree distributions, *Bioinformatics*, Volume 38, Issue 22, 15 November 2022, Pages 5064–5072, https://doi.org/10.1093/bioinformatics/btac630

In the originally published version of this manuscript, the grant number provided in the Funding section was incorrect. The correct grant number is U01 CA227544.

This error has now been corrected.

